# Effects of Prepolymerization and Fly Ash on Exotherm and Flame Retardancy of Polyurethane Mine Grouting Materials

**DOI:** 10.3390/polym18131613

**Published:** 2026-06-29

**Authors:** Rui Feng, Yang Liu, Yuchao Zhang, Jing Zhang, Sitong Zhang, Wenwen Yu, Lan Jia, Qiang Zheng

**Affiliations:** 1 College of Mining Engineering, Taiyuan University of Technology, Taiyuan 030024, China; fengrui@tyut.edu.cn; 2 College of Materials Science and Engineering, Taiyuan University of Technology, Taiyuan 030024, China; liuyang@tyut.edu.cn (Y.L.); zhangyuchao@tyut.edu.cn (Y.Z.); zhangjing@tyut.edu.cn (J.Z.); zhagnsitong@tyut.edu.cn (S.Z.); yuwenwen@tyut.edu.cn (W.Y.)

**Keywords:** prepolymerization, fly ash, polyurethane composite, grouting material, flame retardancy, exotherm control

## Abstract

Conventional polyurethane (PU) grouting materials face a severe trade-off between curing exotherm safety, flame retardancy, and mechanical performance in deep coal mining. Herein, we propose a synergistic strategy combining chemical prepolymerization with fly ash (FA) incorporation to develop high-performance prepolymer-based polyurethane/fly ash (PUP/FA) composite grouting materials. Prepolymerization combined with FA addition successfully mitigated the maximum reaction temperature to 98.3 °C while sustaining a rapid curing rate within 3 min. At an optimal FA loading of 20 wt%, the PUP/FA-20% composite sustained a robust compressive strength of 42.5 MPa, satisfying underground reinforcement standards. Crucially, limiting oxygen index (LOI) and cone calorimetry tests demonstrated outstanding flame retardancy and smoke suppression; the LOI reached 28.5%, and the total smoke production plummeted to 21.3 m^2^. This performance enhancement is governed by a synergistic mechanism where dimethyl methylphosphonate acts via gas-phase radical scavenging, while uniformly dispersed FA particles serve as rigid barrier nodes to construct a dense protective shield in the condensed phase. This work offers a highly effective, waste-valorized, and fire-safe grouting solution for sustainable deep-underground engineering reinforcement.

## 1. Introduction

As coal resource extraction progresses to greater depths, underground engineering faces increasingly complex geological conditions, demanding enhanced tunnel surrounding rock reinforcement technologies and advanced materials [1,2,3]. Polyurethane (PU) grouting materials, distinguished by their exceptional fluidity, rapid curing kinetics, and superior bonding strength, have become highly desirable for coal rock mass consolidation and fracture sealing [4,5,6]. Nevertheless, conventional PU systems exhibit significant drawbacks: intense exothermic reactions during curing cause sharp temperature spikes, leading to performance deterioration, and potential safety hazards in gas- and dust-laden mine environments [5,7,8]. Concurrently, practical grouting applications require robust compressive strength to withstand ground pressure while meeting stringent flame retardancy standards mandated by mining safety regulations [1]. Critically, strategies to mitigate the hazardous exotherm or enhance flame retardancy often compromise mechanical properties, such as compressive strength and elongation, creating a persistent performance trade-off challenge. Therefore, developing novel PU composites capable of simultaneously regulating reaction exotherm, maintaining sufficient mechanical strength, and achieving high flame retardancy holds significant practical importance for enabling safe and sustainable coal mining.

Existing approaches for addressing these issues each have clear advantages but also notable limitations. Controlling exothermic peaks often relies on chemical retarders or reduced catalyst content; while effective at lowering peak temperatures, these approaches typically prolong curing times and compromise early-stage mechanical strength [9,10]. Alternatively, physical methods employing heat-dissipating fillers or phase change materials (PCMs), such as hydrated salts [11], offer thermal regulation. However, despite the ability of PCMs to absorb energy via latent heat, the high loadings required significantly increase viscosity, composite density, and system cost. Regarding flame retardancy, halogenated compounds are efficient but are increasingly restricted due to environmental toxicity concerns [12]. While metal oxide fillers (e.g., aluminum hydroxide) offer a low-cost, non-toxic alternative, their efficacy demands high loadings, which severely impairs fluidity [13,14]. Conversely, phosphorus-based additives are more eco-friendly but often degrade mechanical properties [15]. Therefore, conventional modification strategies fail to overcome the persistent conflict between ensuring thermal safety (exotherm mitigation and flame retardancy) and maintaining optimal mechanical performance.

Prepolymerization, an established PU synthesis technique widely employed in waterborne systems, offers a promising pathway for exotherm control [16,17]. This method involves prereacting excess isocyanate with polyols to form terminal -NCO prepolymers, effectively reducing the system’s exothermic temperature [18,19]. Separately, fly ash (FA), an abundant, low-cost coal combustion byproduct, presents opportunities for solid waste valorization and multifunctional material modification [20,21,22]. By repurposing this industrial waste as a grouting material, FA utilization addresses both engineering safety and ecological risks, aligning with green and sustainable mining practices. Prior studies suggest that FA incorporation into PU matrices can improve flame retardancy and thermal stability [23,24,25]. While prepolymerization offers active thermal management and FA provides improved flame retardancy, the synergistic potential of combining prepolymerization with optimized FA loading to balance exotherm control, mechanical integrity, and flame retardancy, specifically targeting the stringent requirements of mining grouts, remains underexplored.

Herein, we propose a synergistic modification strategy combining prepolymerization with controlled FA incorporation to develop high-performance prepolymer-based polyurethane (PUP/FA) composite grouting materials. A series of PUP/FA composites with varying FA loadings were fabricated via room-temperature curing. The microstructure was analyzed using scanning electron microscopy (SEM). Furthermore, we evaluate their synergistic effects on system viscosity, peak reaction temperature, curing time, mechanical strength, thermal stability, and flame retardancy. This work establishes a foundation for designing advanced PU mining grouts where the combined benefits of prepolymerization and FA incorporation can overcome the typical performance trade-offs, ultimately yielding the grouting materials achieving a compressive strength of 42.5 MPa, a peak reaction temperature of 98.3 °C, and an LOI of 28.5%, meeting critical safety and performance benchmarks.

## 2. Experimental Section

### 2.1. Materials

Industrial-grade polyether polyols 305 (average molecular weight Mn ≈ 500), 330 (Mn ≈ 3000), and PPG-400 (Mn ≈ 400) were supplied by Nantong Ruitai Chemical Co., Ltd. (Haian, China). Dibutyltin dilaurate (DBTDL, industrial grade) was obtained from Shanghai Aladdin Biochemical Technology Co., Ltd. (Shanghai, China), and polymethylene polyphenyl isocyanate (PAPI, industrial grade) was supplied by Shandong Yantai Wanhua Co., Ltd. (Yantai, China). FA (300 mesh, industrial grade) was purchased from Henan Bolun Casting Materials Co., Ltd. (Luoyang, China). Dimethyl methylphosphonate (DMMP, industrial grade), chlorinated paraffin (industrial grade), and foam stabilizer AK-158 (industrial grade) were provided by Shenzhen Dianshifang Technology Co., Ltd. (Shenzhen, China), Guangzhou Fufei Chemical Technology Co., Ltd. (Guangzhou, China), and Shandong Aolilong Chemical Co., Ltd. (Tengzhou, China), respectively. For the dibutylamine titration method, analytical-grade reagents bromothymol blue indicator, concentrated hydrochloric acid, n-butylamine, and acetone, were sourced from Shanghai Mclean Biochemical Technology Co., Ltd. (Shanghai, China) and China National Pharmaceutical Group Chemical Reagents Co., Ltd. (Shanghai, China). Prior to use, polyether polyols and FA were vacuum-dried at 120 °C to remove residual moisture. All other raw materials were used directly.

### 2.2. Preparation of PU/FA Grouting Composites

#### 2.2.1. Preparation of Prepolymerized Polyurethane (Pre-PU) and Component B

An appropriate amount of PPG-400 was added to a prepolymerization container and dried in a vacuum oven at 110 °C for 2 h. After cooling to room temperature, a specified amount of PAPI was added, and the mixture was stirred at 500 rpm on a magnetic stirrer at 500 rpm for 10 min. The homogenized mixture was then reacted under a nitrogen atmosphere at 80 ± 2 °C for 2 h to obtain the prepolymer component. Once cooled to room temperature, flame retardants (DMMP and chlorinated paraffin) were added, and the mixture was thoroughly stirred at 500 rpm for 10 min using a mechanical stirrer to obtain the required prepolymer B component. The -NCO content of the prepared prepolymer was 26.85% measured by a dibutylamine titration method [17].

#### 2.2.2. Preparation of PUP/FA Grouting Material by the Prepolymer Method

**Figure 1:**
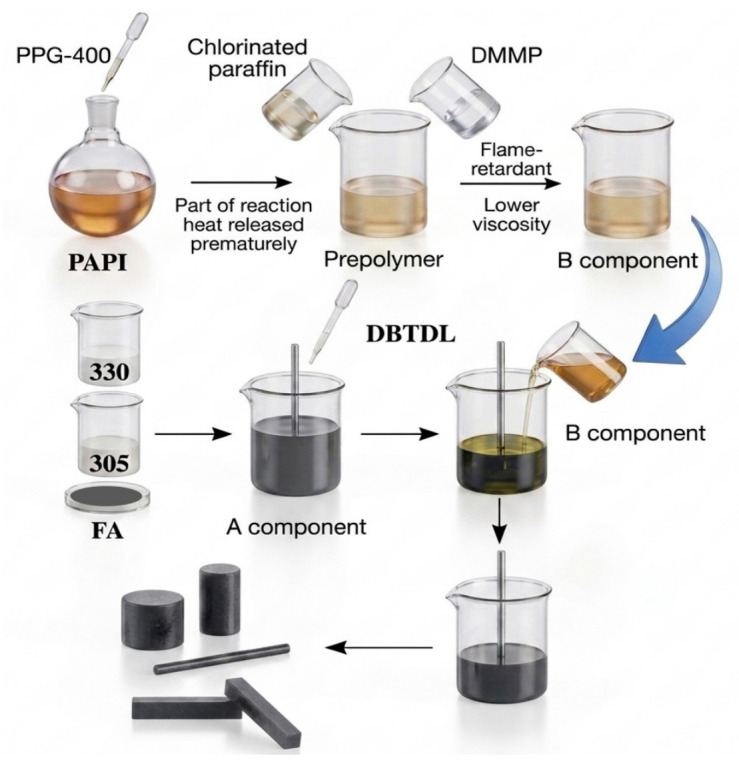
Schematic diagram of the PUP/FA preparation.

The preparation process for the PUP/FA grouting material is shown in Figure 1. A suitable amount of FA was weighed and dried at 120 °C in a vacuum drying oven for 2 h before being sealed for storage. A specified amount of polyether polyol 330, the dried FA, AK-158, and DBTDL were added to a cup containing polyether polyol 305, and the mixture was thoroughly stirred at 500 rpm to form component A. When the prepolymer (component B) cooled to 23 ± 2 °C, components A and B were mixed quickly at 500 rpm and poured into different molds. After curing, the PUP/FA composite material was obtained.

#### 2.2.3. Preparation of PU/FA Grouting Material by the One-Step Method

FA was weighed and dried at 120 °C in a vacuum drying oven for 2 h before being sealed for storage. A specified amount of PPG-400, polyether polyol 330, the dried FA, AK-158 and DBTDL were added to a cup containing polyether polyol 305, and mixed thoroughly at 500 rpm to form component A. A specified amount of isocyanate, flame retardants (DMMP and chlorinated paraffin) were weighed and mixed thoroughly at 500 rpm to form component B. At 23 ± 2 °C, components A and B were mixed quickly at 500 rpm and poured into prepared molds. After curing, the PU/FA composite material was obtained.

The experimental procedure fixed the R value (R = n(-NCO)/n(-OH)) at 1.31, where n(-OH) represent the total moles of hydroxyl groups from polyether polyols 305, 330, and PPG-400. And the catalyst amount of DBTDL was set at 0.16 g. The specific formulations of PUP/FA composite material are shown in Table 1. The PU/FA composite was prepared using an identical formulation.

### 2.3. Characterization

The infrared spectra of polyol PPG-400, isocyanate PAPI, and the prepared prepolymer were measured using an infrared spectrometer (Invenio S, Bruker, Mannheim, Germany) in the scanning range of 500–4000 cm^−1^.

Under conditions of 23 ± 2 °C, 200 mL samples of components A and B were prepared, and the viscosity of PU/FA and PUP/FA composite materials was measured using a viscometer (NDJ-5S, Shangpu Instrument, Shanghai, China).

Following the AQ/T 1089-2020 standard [26], 200 mL of the test sample was prepared according to the formula. After stirring uniformly for 15–20 s, the mixture was poured into a cylindrical container with a 50 mm diameter, and a thermocouple was placed in the center of the sample to measure the maximum reaction temperature.

In accordance with the AQ/T 1089-2020 standard, 200 mL of the sample consisting of components A and B was prepared at a temperature of 20 °C. The sample was poured into a cylindrical container with a 50 mm diameter, and mechanical stirring was performed at 500 rpm while the time was recorded. The timer was stopped once the surface of the material just began to cure, and the time interval was recorded.

According to the AQ/T 1089-2020 standard, cylindrical samples with a diameter of 50 mm and height of 100 mm were prepared with different filler contents. The compressive strength of the samples was tested using a universal testing machine (Z25003051242, Zwick Roell, Ulm, Germany).

PU/FA and PUP/FA composite material samples were prepared, immersed in liquid nitrogen for brittle fracture, and their fracture morphology was observed using SEM (Gemini SEM 360, Zeiss, Oberkochen, Germany).

The LOI of PU/FA and PUP/FA composite materials was tested according to the GB/T 2406.2-2009 standard [27] using a critical oxygen index instrument (PX-01-005, PHINIX, Nanjing, China) to investigate the combustion behavior of the materials under self-ignition conditions. All experiments were repeated five times, and the results are expressed as the mean.

The thermal stability of PU/FA and PUP/FA composite materials was tested under a nitrogen atmosphere using a TG analyzer (NETZSCH TG-209, NETZSCH, Helb, Germany) over a temperature range of 30–900 °C with a heating rate of 10 °C/min.

The X-ray diffraction patterns were obtained using CuKα radiation (1.5418 Å) in X-ray diffractometer (XRD, MAX-2600, Rigaku D, Tokyo, Japan) to study the effect of the fly ash additives on the structure of the PU grouting composite.

The conical calorimetry was performed to test the combustion performance of the composite grouting materials. A cone calorimeter (FTT-0242, FTT, East Grinstead, UK) was used for the conical calorimetry in accordance with ISO 5660-1-2002 [28]. The heat flux was 35 kW/m^2^.

## 3. Results and Discussion

### 3.1. FT-IR Analysis of Prepolymer

**Figure 2:**
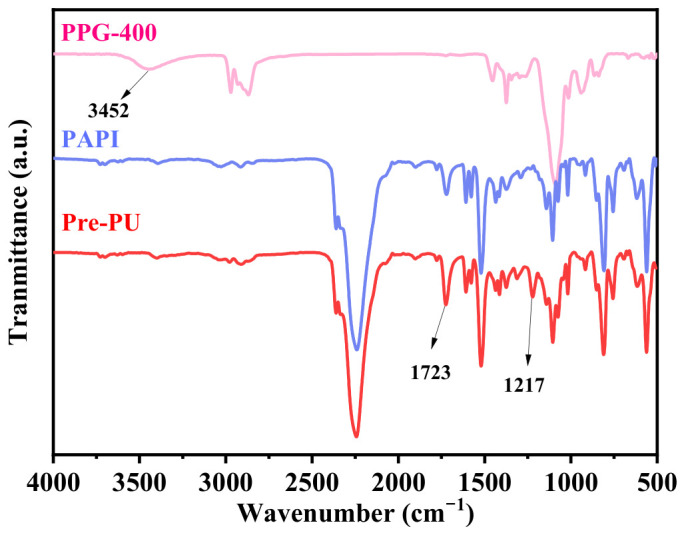
Fourier transform infrared spectroscopy of PAPI, PPG-400 and pre-PU.

To confirm the successful preparation of the pre-PU, the chemical structure of PAPI, PPG-400 and pre-PU was characterized using FT-IR, as shown in Figure 2. Compared to polyol, the peak intensity of the -OH stretching vibration of pre-PU at 3452 cm^−1^ decreased significantly, while a new stretching vibration peak corresponding to the C-O bond of the carbamate group emerged at 1217 cm^−1^, confirming the formation of carbamate bonds [29,30]. In addition, the characteristic absorption peak of the -NCO carbonyl group at 1723 cm^−1^ remained after prepolymerization, indicating that the prepolymer still possesses reactivity with polyol. The -NCO content of the prepared prepolymer was determined to be 26.85%.

### 3.2. Viscosity of the Components A and B

**Figure 3:**
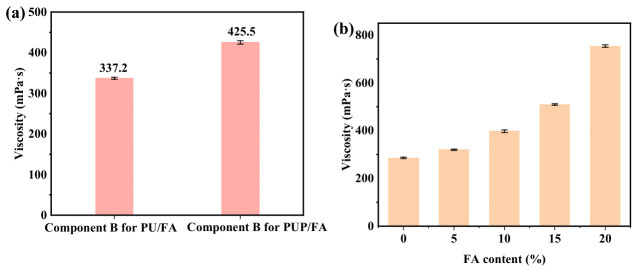
Variations in the viscosity change in (**a**) components B and (**b**) components A.

The viscosities of components A and B in the PU/FA and PUP/FA composite materials were measured using a viscometer, and the results for viscosity changes under different filler contents are shown in Figure 3. The viscosities of the components B for PU/FA and PUP/FA were 337.2 mPa·s and 425.5 mPa·s, respectively. The increased viscosity of the component B for PUP/FA is attributed to the reaction between isocyanate and a small amount of polyol during the prepolymerization process, which results in an increase in the molecular weight of the prepolymer [31,32]. In addition, the flame retardants (DMMP and chlorinated paraffin) also contribute to maintaining an advantageous low viscosity in the system. Furthermore, since FA is present in component A, the viscosity of the component A in both PU/FA and PUP/FA composites increased significantly as the FA content increased. When the FA content reached 20%, the viscosity of the component A increased to 754.3 mPa·s. Nevertheless, all viscosity values remained within the acceptable range for grouting applications.

### 3.3. Maximum Reaction Temperature and Curing Time for Grouting Materials

**Figure 4:**
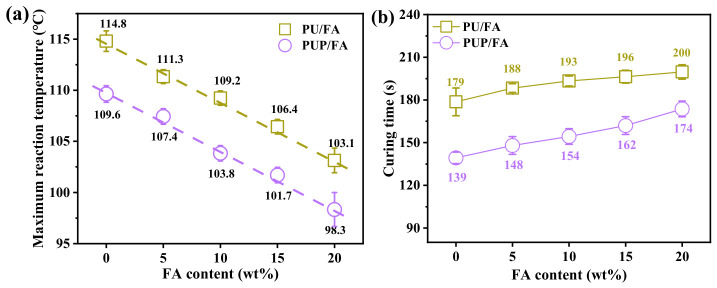
(**a**) The maximum reaction temperatures and (**b**) curing time of PU/FA and PUP/FA with different FA contents.

To investigate the effect of prepolymerization on the thermal curing behavior of PU/FA composites, the maximum reaction temperatures and curing times were measured under identical catalyst dosages, as shown in Figure 4.

The maximum reaction temperature of pure PUP was 109.6 °C, which is 4.5% lower than that of pure PU (114.8 °C). This reduction indicates that prepolymerization enables partial reaction between isocyanate (-NCO) and hydroxyl (-OH) groups during the synthesis stage, preemptively releasing a portion of the reaction heat. Consequently, the subsequent exothermic heat released during the crosslinking with polyether polyols is reduced, thereby lowering the maximum reaction temperature of the PUP system. Notably, the PUP/FA system consistently exhibited a significantly lower maximum reaction temperature than the PU/FA system, with a temperature difference of approximately 4~5.5 °C. With the addition of FA, the maximum reaction temperatures of both composites decreased linearly. When the FA content reached 20 wt%, the temperatures for PU/FA and PUP/FA dropped to 103.1 °C and 98.3 °C, respectively, representing reductions of ~11.5 °C compared to their pure counterparts.

As depicted in Figure 4b, the curing time of pure PUP was 139 s, notably shorter than the 179 s observed for pure PU. This suggests that prepolymerization accelerates the curing process. Consistently, the curing time of the PUP/FA system was significantly shorter than that of the PU/FA system across all compositions. As the FA content increased, the curing times of both composites showed a monotonically increasing trend. At 20 wt% FA, the curing times for PU/FA and PUP/FA reached their maximum values of 200 s and 174 s, respectively. The prolonged curing time is attributed to the FA filler acting as a diluent, which lowers the local concentration of -NCO and -OH groups in the polyurethane matrix, thereby reducing the heat release per unit volume. Furthermore, the FA particles raises the system viscosity (Figure 3b) and impedes the diffusion and collision of molecular chains, thereby prolonging the curing time [33]. Despite the extension caused by FA, the prepolymerization strategy imparts the PUP/FA composites with superior curing performance, balancing a reduced maximum reaction temperature (98.3 °C) with rapid curing rates (under 3 min), making the PUP/FA-20% composite highly suitable for the rapid reinforcement requirements of mining applications.

### 3.4. Mechanical Properties

**Figure 5:**
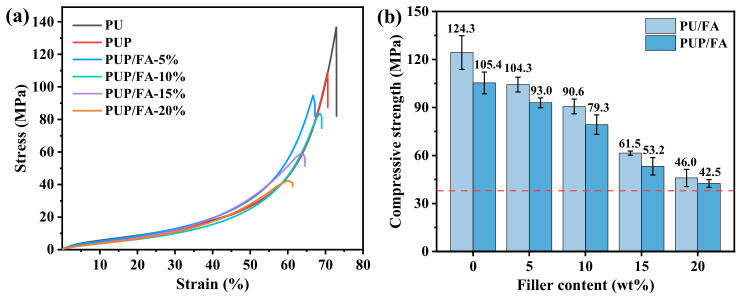
(**a**) The stress–strain curves and (**b**) the compression strength of PU grouting materials. The dotted line at 40 MPa represents the mandatory minimum compressive strength specified in AQ/T 1089-2020.

Figure 5 illustrates the stress–strain curves and the compressive strength of the PU grouting materials. A distinct difference is observed between pure PU and PUP, where the compressive strength of pure PUP is slightly lower than that of pure PU. This phenomenon can be attributed to the differences in crosslinking density [34]; unlike the one-step method which constructs a high-density crosslinked network directly via the reaction of high molecular weight polyols with excess isocyanate, the PUP approach utilizing chain extenders results in a relatively lower crosslinking density [35], thereby compromising mechanical strength. With the increase in FA content, the compressive strength of both PU/FA and PUP/FA composite materials gradually decreased, and the elongation at break of PUP/FA composites also exhibited a declining trend (Figure 5a). Notably, the compressive strength of PUP/FA composites remained significantly lower than that of PU/FA composites across all FA loadings (Figure 5b). Under high filler loading, the FA particles act as stress concentration points within the matrix, where stress tends to accumulate at the particle-matrix interface [36,37]. This not only disrupts the integrity of the continuous PU phase and induces compressive failure but also impedes the cooperative deformation of the PU molecular chains due to the rigid nature of FA, thereby reducing the compressive compliance of the system. Despite these reductions, when the FA content reached 20%, the compressive strengths of the PU/FA and PUP/FA composite grouting materials were 46.0 MPa and 42.5 MPa, respectively. These values are well within the requirement for mining polymer reinforcement materials, which mandate a compressive strength greater than 40 Mpa, according to the AQ/T 1089-2020 (industry standard for safety production in China). Although both prepolymerization and FA addition lead to a decrease in compressive strength, the incorporation of 20 wt% FA represents the optimal compromise between mechanical performance, safety, and processability for mine grouting applications.

### 3.5. Morphological and Structural Characterization

**Figure 6:**
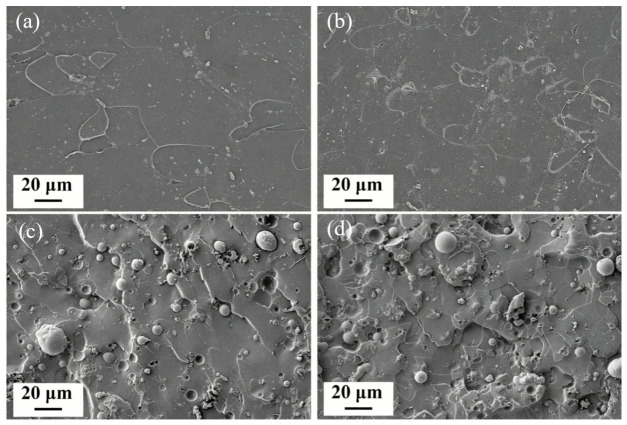
SEM images of (**a**) PU, (**b**) PUP, (**c**) PU/FA-20%, and (**d**) PUP/FA-20%.

The SEM images of the cross-sections of PU, PUP, PU/FA-20%, and PUP/FA-20% composite materials are shown in Figure 6, respectively. The cross-sections of the pure PU and PUP samples appear relatively smooth and flat, displaying predominantly smooth river-like cracks, which is indicative of a typical brittle fracture morphology [38]. In contrast, the cross-section of the PU/FA-20% composite material exhibits obvious spherical protrusions, and the interfacial bonding between the FA and the matrix is loose, characterized by noticeable pores resulting from FA pull-out. The FA particles achieve a micron-level uniform dispersion within the polyurethane matrix, with single particle sizes primarily distributed in the range of 1–10 μm. The cross-section of the PUP/FA-20% composite material presents characteristics similar to those of the PU/FA-20% composite, featuring distinct spherical protrusions and small wrinkles. This suggests that the prepolymerization process does not alter the dispersion state of the FA filler. Notably, the SEM images reveal poor adhesion between the FA particles and the polyurethane matrix, as evidenced by gaps generated upon particle pull-out. These areas of weak interfacial bonding act as stress concentration points, which explains the observed decrease in compressive strength with increasing FA content.

**Figure 7:**
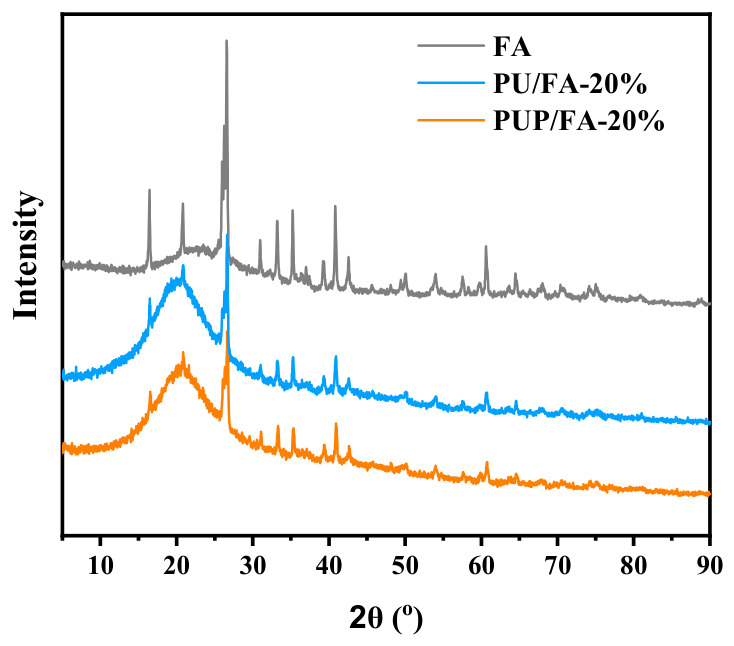
XRD analysis of FA and PU composite materials.

To further investigate the phase structure and dispersion state of the components, XRD characterization was conducted on pristine FA, PU/FA, and PUP/FA composites, as illustrated in Figure 7. The XRD analysis detected mullite and quartz as major sharp peaks within the crystalline fly ash particles, which consist primarily of silica and alumina [39]. Meanwhile, both the PU/FA and PUP/FA composites display a broad, diffuse halo characteristic of amorphous molecular chains. With the addition of FA, the sharp crystalline reflections of FA (most notably the dominant quartz peak at 2θ = 26.6°) are clearly superimposed. This observation indicates that the FA is physically dispersed within the polymer matrix without any change in its intrinsic FA crystal structure.

### 3.6. Flame Retardant Performance of the Composite Materials

**Figure 8:**
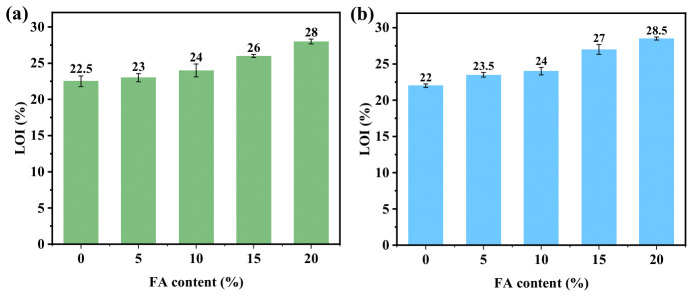
LOI value of PU grouting materials: (**a**) PU/FA and (**b**) PUP/FA.

Flame retardancy is a critical requirement for PU grouting materials in coal mine applications. The LOI values of the PU/FA and PUP/FA composite materials are presented in Figure 8. Both pure PU and PUP are inherently combustible, exhibiting LOI values of 22.5 and 22, respectively. However, with increasing FA content, the LOI values of both composites gradually improved. When the FA content reached 20%, the LOI values of PU/FA-20% and PUP/FA-20% reached their maximums of 28 and 28.5, respectively, achieving a classification of difficult to ignite. This indicates that the incorporation of FA effectively enhances the flame retardancy of the composite materials. A comparison of the two systems reveals that the difference in LOI values between PU/FA and PUP/FA is minimal. This negligible change can be attributed to the constant flame retardant dosage in both formulations; thus, the prepolymerization process does not significantly alter the flame retardancy of the composites. While prepolymerization increases the viscosity of the system, the resulting microscopic morphological changes are not significant enough to influence the overall flame retardant performance.

**Figure 9:**
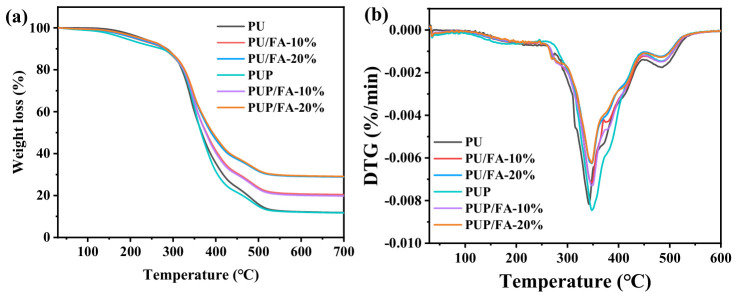
(**a**) TGA and (**b**) DTG of PU/FA and PUP/FA.

To further investigate the effects of prepolymerization and FA content on the thermal stability of the composites, TG and DTG analyses were conducted under a nitrogen atmosphere, as illustrated in Figure 9, with the corresponding thermogravimetric data listed in Table 2. The PU/FA composites exhibit a three-stage decomposition process: thermal loss below 300 °C is primarily attributed to the volatilization of the added flame retardants, DMMP and chlorinated paraffin; decomposition between 300–450 °C corresponds to the degradation of carbamate groups and the scission of polyol long chains; and further breakdown of the PU soft segments occurs between 450–550 °C [40,41].

For PUP/FA composites, the thermal degradation profile follows a similar three-stage pattern. However, as shown in Figure 9 and Table 2, the initial decomposition temperature of PUP is slightly lower than that of PU, indicating that prepolymerization marginally reduces the thermal stability of the system. This reduction is likely attributable to the increased structural defects induced by the prepolymerization process. Despite this, the temperatures corresponding to the three weight loss peaks in both PU/FA and PUP/FA composites remain largely consistent. Notably, with increasing FA content, the *T*_50%_ decomposition temperature of PU/FA composites rises from 366 °C to 389 °C, while that of PUP/FA composites increases from 365 °C to 393 °C. This significant enhancement suggests that the oxides present in FA facilitate act as solid-acid catalysts that promote the dehydrative carbonization and crosslinking of the degrading polyurethane chains, thereby forming a dense, highly cohesive carbonaceous-inorganic hybrid char residue (as evidenced higher final residual mass compared to PU or PUP) during thermal decomposition [42,43,44]. Physically, this robust hybrid char layer, combined with the thermally stable silica-alumina network of the FA, serves as an effective thermal and mass barrier, obstructing heat transfer and suppressing the pyrolysis of the underlying polymer matrix, thereby increasing both the decomposition temperature and the final residual mass of the composites.

When the FA content is less than 20 wt%, the composite cannot form a sufficiently dense barrier, resulting in a LOI below 27.0%, which fails the safety threshold for underground mines. Conversely, when the FA loading exceeds 20 wt%, the viscosity of the liquid system surges beyond 1000·mPa·s, severely hindering pumpability and micro-fissure penetration under practical grouting conditions, while the compressive strength deteriorates below 40.0 MPa due to excessive dilution of the polyurethane matrix. Notably, at 20 wt% FA, the composite achieves a critical balance: a low maximum reaction temperature of 98.3 °C, LOI 28.5%, and a high compressive strength of 42.5 MPa. All these parameters successfully meet the requirements of the coal mine industry standard AQ/T 1089-2020, confirming that 20 wt% FA is the optimal formulation. To demonstrate the overall performance superiority of the developed PUP/FA-20% composite, a comparative analysis with previously reported modified polyurethane systems was conducted (as shown in Table 3). The comparative results highlight that the PUP/FA-20% composite exhibits a superior balance of maximum reaction temperature, LOI, and compressive strength.

**Figure 10:**
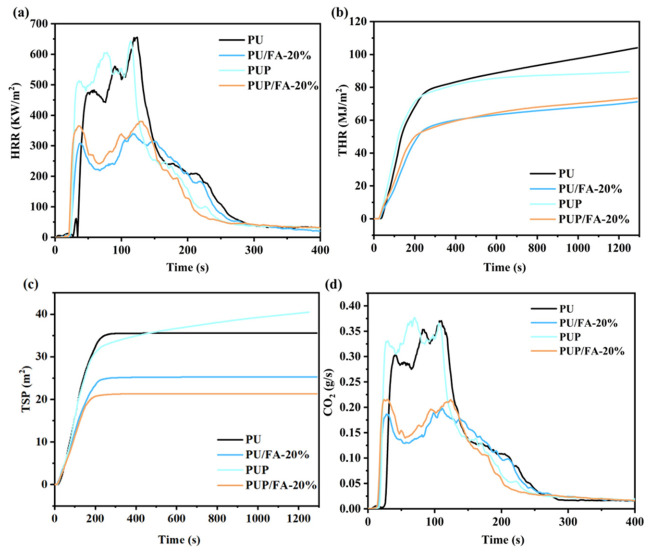
(**a**) The heat release rate, (**b**) the total heat release, (**c**) the total smoke production, and (**d**) CO_2_ release rate curves of PU/FA, PUP/FA, PU/FA-20% and PUP/FA-20%.

To further evaluate the flame-retardant performance of the PU composites, cone calorimetry tests were performed, as illustrated in Figure 10. Figure 10a presents the heat release rate (HRR) of pristine PU, the prepolymerized PUP and their composites. Pristine PU exhibits the highest peak HRR, while the PUP matrix displays slightly faster initial combustion kinetics but a comparable maximum peak. Upon the incorporation of 20 wt% FA, the HRR curves of both matrices are significantly suppressed, accompanied by a rapid decay in heat release at the later stages of combustion. Consistent with the HRR trends, the total heat release (THR) curves (Figure 10b) demonstrate that pristine PU reaches a final THR of 104.1 MJ/m^2^, which decreases to 89.4 MJ/m^2^ for PUP. With the addition of 20 wt% FA, the THR values are further suppressed to 71.2 MJ/m^2^ (PU/FA-20%) and 73.4 MJ/m^2^ (PUP/FA-20%), indicating that FA effectively dilutes the combustible organic components and facilitates char yield. Moreover, smoke and CO_2_ emissions are also significantly mitigated (Figure 10c,d). It is noted that the total smoke production (TSP) of PUP (40.8 m^2^) decreases to 21.3 m^2^ (PUP/FA-20%), demonstrating outstanding smoke suppression. Similarly, the CO_2_ release rate curves exhibit a double-peak profile, with the peak values dropping from 0.37–0.38 g/s for unfilled matrices to approximately 0.20–0.21 g/s for the FA-loaded composites, indicating a significantly inhibited combustion reaction rate.

These remarkable enhancements in flame retardancy and smoke suppression are attributed to the synergistic mechanism between the PU matrix, DMMP, chlorinated paraffin, and FA. During thermal decomposition, chlorinated paraffin releases non-combustible gases at relatively lower temperatures, which dilute the oxygen and flammable volatiles in the flame zone. Simultaneously, DMMP decomposes to generate active phosphorus-containing radicals. These radicals act as high-efficiency radical scavengers that capture highly reactive H· and OH· radicals in the gaseous combustion zone, terminating the chain reaction of flame propagation [15]. In the condensed phase, acidic phosphorus species generated by DMMP decomposition catalyze the esterification, dehydration, and carbonization of the polyether chains in the PU matrix. This process accelerates the formation of an initial carbonaceous char barrier. The FA particles, primarily consisting of thermally stable silica (SiO_2_) and alumina (Al_2_O_3_), act as physical barrier nodes. During combustion, as the polymer matrix retreats, these uniformly dispersed rigid FA particles integrate with the carbonaceous char catalyzed by DMMP to form a continuous, dense, and robust silicon-aluminum (Si-Al) rich inorganic–organic hybrid protective shield [20]. This compact shield effectively restricts heat transfer, insulates oxygen, and prevents the escape of volatile pyrolysis gases, leading to the observed reductions in HRR, THR, and TSP. Specifically for the PUP/FA-20% composite, the faster initial combustion kinetics of the PUP matrix, combined with the catalytic dehydration of acidic phosphorus species from DMMP decomposition, accelerates the formation of a carbonaceous char cover at the early stages of combustion. This timely char layer traps smoke-generating volatiles before they can enter the flame, resulting in the material’s great smoke suppression.

## 4. Conclusions

In summary, the prepolymer-based component B was successfully prepared using PAPI, PPG-400, DMMP, and chlorinated paraffin. PUP/FA composite grouting materials were then synthesized via room temperature curing. The effects of prepolymerization and FA content on the maximum reaction temperature, curing time, mechanical properties, thermal stability, and flame retardancy of PU/FA composite grouting materials were thoroughly investigated. The main conclusions are as follows:

(1) The viscosity of the prepolymer B component increased from 337.2 mPa·s to 425.5 mPa·s after prepolymerization. Additionally, the viscosity of component A was found to increase with higher FA content. Both prepolymerization and the addition of FA reduce the maximum reaction temperature of the grouting materials, with prepolymerization additionally shortening the curing time. This achieves a favorable balance between a mitigated maximum reaction temperature (98.3 °C) and a rapid curing rate (within 3 min), which is beneficial for grouting operations.
(2) Compared to pure PU and PU/FA composites, the compressive strength of PUP and PUP/FA composites is slightly lower due to reduced crosslinking density. Despite a gradual decrease with increasing FA content, the strength remains above 42.5 MPa at 20 wt% FA, which is considered adequate for typical coal rock reinforcement applications.
(3) LOI and TG analyses showed that prepolymerization reduces the initial pyrolysis temperature of the composites, while the addition of FA increases the residual mass and suppresses the pyrolysis rate. Notably, the LOI of PUP/FA composite grouting materials significantly rises with increasing FA content. When the FA content reaches 20%, the LOI is 28.5%. Furthermore, incorporation 20 wt% FA significantly suppressed the heat release rates of both systems.

Although current physical blending methods meet requirements, future chemical modifications will be examined to improve the interfacial bonding, enabling higher waste loading while maintaining mechanical property. Additionally, research will explore combining other industrial by-products, including slag and red mud, to leverage synergistic effects for improved performance and sustainability.

## Figures and Tables

**Table 1:** Formulations of the PU/FA composites.

Sample	PAPI (g)	DMMP (g)	Chlorinated Paraffin (g)	PPG-400 (g)	305 (g)	330 (g)	AK-158 (g)	FA (g)
PUP	36	5	8	3	29	18	1	0
PUP/FA-5%	34.2	4.75	7.6	2.85	27.55	17.1	0.95	5
PUP/FA-10%	32.4	4.75	7.2	2.7	26.1	16.2	0.9	10
PUP/FA-15%	30.6	4.25	6.8	2.55	24.65	15.3	0.85	15
PUP/FA-20%	28.8	4	6.4	2.4	23.2	14.4	0.8	20

**Table 2:** Thermogravimetric data of PU/FA and PUP/FA composites.

Sample	T_5_% (°C)	T_50_% (°C)	Peak Temperature of the First Stage (°C)	Peak Temperature of the Second Stage (°C)	Peak Temperature of the Third Stage (°C)	Final Residual Mass (%)
PU	228	366	215	342	486	12.3
PU/FA-10%	211	378	214	348	488	20.8
PU/FA-20%	219	389	215	347	486	29.6
PUP	188	365	210	348	486	11.9
PUP/FA-10%	222	376	214	349	487	19.9
PUP/FA-20%	225	393	215	349	485	29.7

**Table 3:** Comparison of key performance of the PUP/FA composite with other polyurethane-based systems in the literature.

Grouting Material	Maximum Reaction Temperature (°C)	LOI (%)	Compressive Strength (MPa)
This study	98.3	28.5	42.5
PU/FA-20% [23]	125.0	26.2	32.0
PU/FA-15%/LDH-5% [23]	110.0	27.5	37.5
PU/FA-20%/WG [25]	115.0	-	57.0
PU/Al_2_O_3_-WG [45]	100.5	-	36.8
PU/EG/SAT [7]	80.6	-	0.45

## Data Availability

The original contributions presented in the study are included in the article; further inquiries can be directed to the corresponding author.
